# Supplementary material for: Magmas near the critical degassing pressure drive volcanic unrest towards a critical state
Source: Nat Commun. 2016 Dec 20;7:13712. doi: 10.1038/ncomms13712 (PMC5187427; doi:10.1038/ncomms13712)
Supplement: Supplementary Information — Supplementary Figures and Supplementary Table. [file ncomms13712-s1.pdf]

Supplementary Table 1. Timing, CO_2/H_2O molar ratio, and total mass of the simulated episodes of IMF.

		Simulation 1		Simulation 2	
Injection number	Month and year	CO_2/H_2O	Mass (Mt)	CO_2/H_2O	Mass (Mt)
1	October 1983	0.67	6.55	0.67	6.55
2	July 1988	0.67	3.39	0.61	3.04
3	September 1993	0.67	1.24	0.47	1.45
4	May 1999	0.67	0.45	0.35	0.61
5	April 2000	0.67	0.11	0.33	0.31
6	August 2001	0.67	0.11	0.31	0.32
7	November 2002	0.67	0.79	0.29	1.50
8	July 2005	0.67	2.03	0.26	3.57
9	August 2006	0.67	3.05	0.25	8.76
10	November 2007	0.67	0.68	0.24	2.24
11	January 2009	0.67	2.82	0.24	9.55
12	May 2010	0.67	1.47	0.23	9.72
13	October 2011	0.67	2.26	0.23	12.99
14	November 2012	0.67	4.97	0.22	25.73

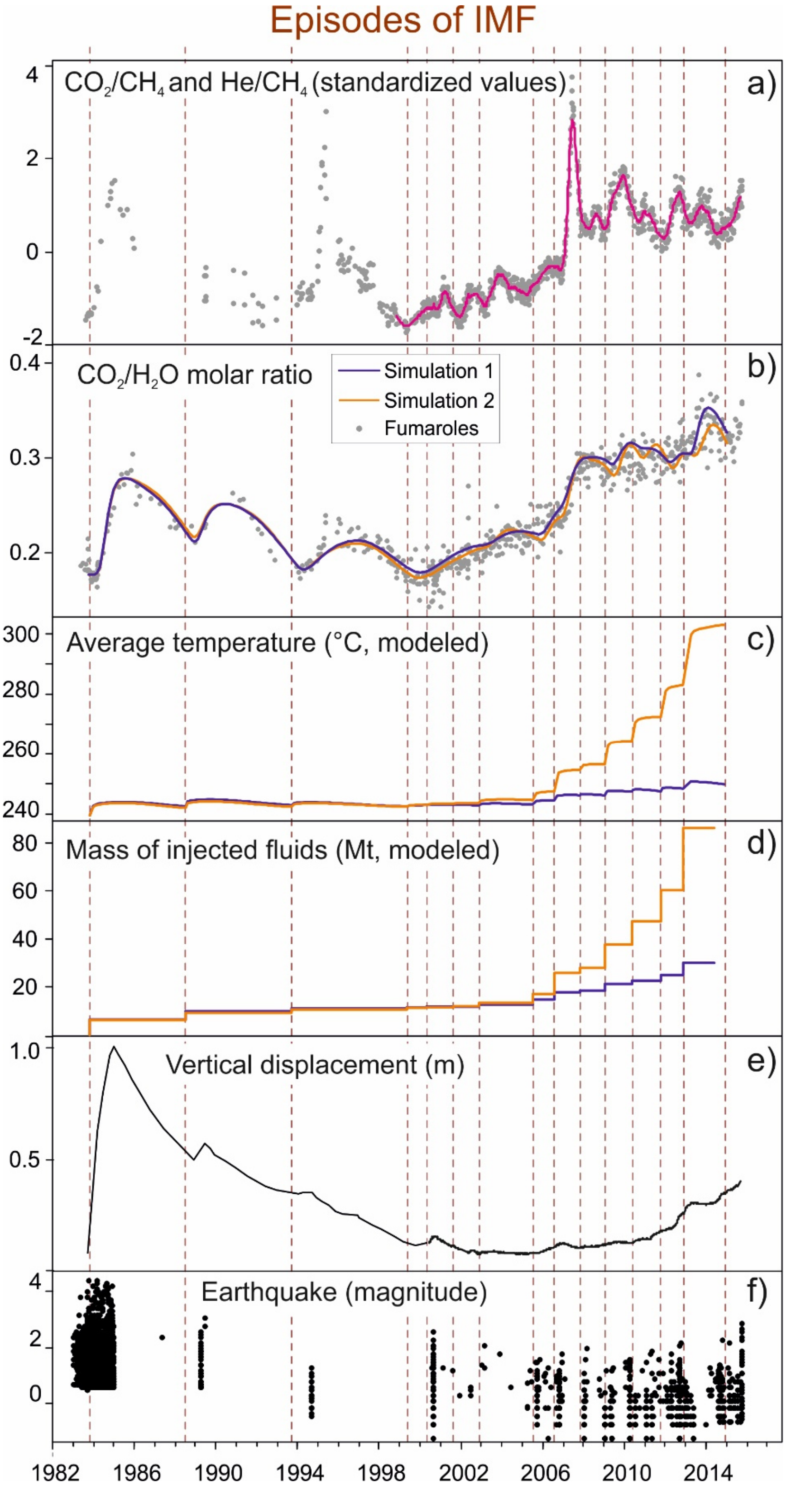

Supplementary Figure 1. Time series of observed data and model results. (a) Measured CO_2/CH_4 and He/CH_4 ratios at Solfatara fumaroles. In order to compare the different signals, data were normalized (standardized *z* score) by removing the mean and dividing by the standard deviation. The magenta curve shows the 4-month moving average for the data. (b) Measured CO_2/H_2O ratio at Solfatara fumaroles (gray dots) compared with the value simulated in the "checkpoint for gas composition" (see Fig. 4). (c) Average temperature in the central deep zone of the computational domain for the two simulations. (d) Cumulative total mass of modeled injected fluids. (e) Maximum vertical displacement during 1984–2016 (CGPS data after 2000, leveling data before 2000). (f) Magnitudes of earthquakes during 1982–2016. The timing of each magmatic fluid injection episode (IMF) is indicated by brown dashed lines
.

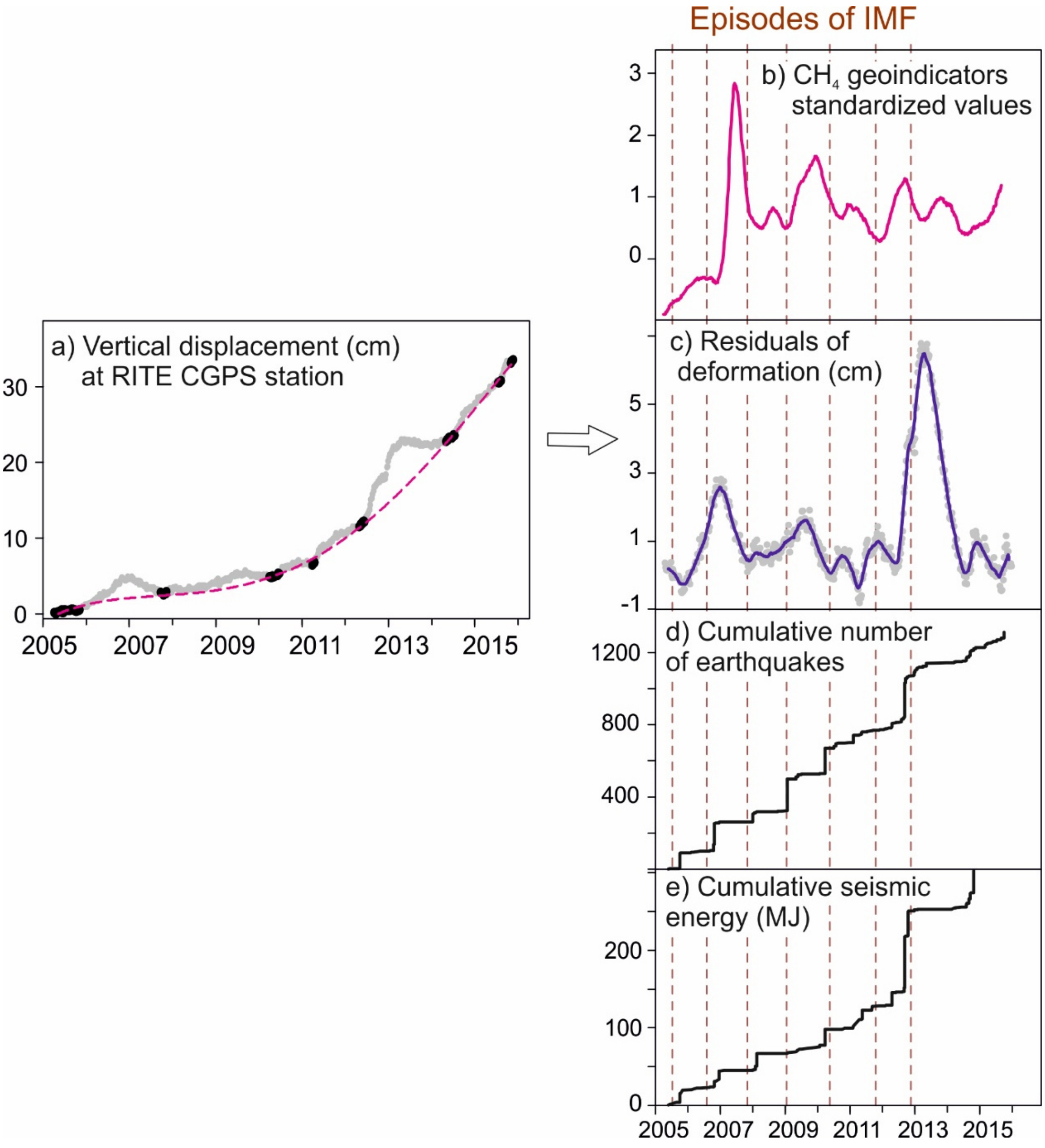

Supplementary Figure 2. Evolution of monitoring signals at CFc during the current unrest (post-2005). (a) Vertical displacements at RITE CGPS station during 2005–2015. Black dots indicate the data used to derive a polynomial fit of the accelerating trend curve[14] (dashed magenta line). (b) Four-month moving average of CH_4-based geoindicators (see Supplementary Fig. 1). (c) Residual of observed ground deformation (dots in panel a) with respect to the fitted model (magenta line in panel a). (d) Cumulative number of earthquakes during 2005–2014. (e) Cumulative seismic energy released during 2005–2014. The timing of each magmatic gas injection episode (brown dashed lines) is assumed to be 300 days before the geochemical peaks. Concurrent ground deformation pulses (panel c) and earthquakes (panels d, e) strongly support the timing of the magmatic gas injection events.

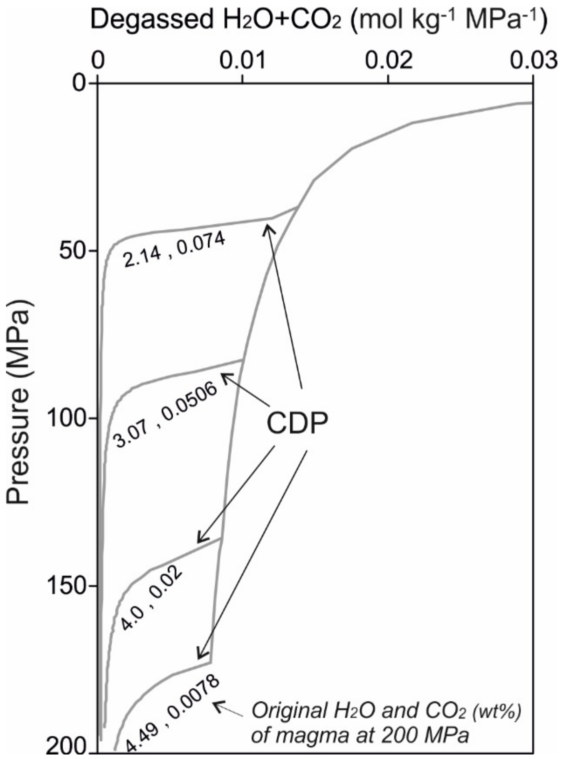

Supplementary Figure 3. Open-system magma-degassing models for basalts (SiO_2 = 49 wt%) using VolatileCalc code[36]. The different curves refer to different initial H_2O and CO_2 contents and describe the evolution of the fluids released during magma depressurization in an open-system Rayleigh-type degassing process (where at each infinitesimal decompression step, an infinitesimal parcel of gas phase in excess of the permissible saturation is distilled from the well-mixed magma).

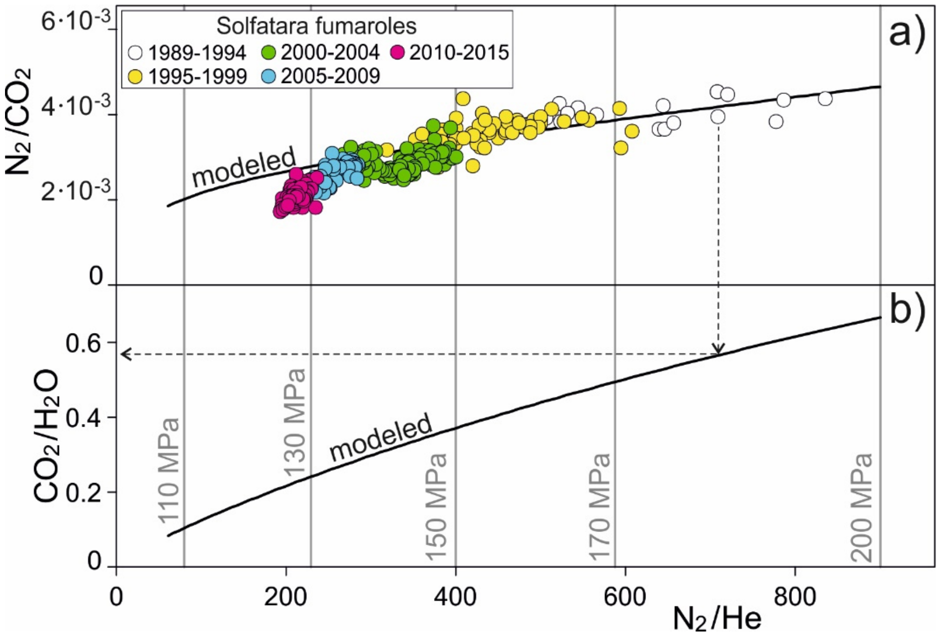

Supplementary Figure 4. (a) Measured N_2/He and N_2/CO_2 fumarole ratios compared with the modeled compositions of the fluids released by depressurizing open-system degassing of a trachybasalt magma[21]. Assuming an initial pressure of 200 MPa, and an initial gas phase characterized by N_2/He and N_2/CO_2 ratios of 900 and 0.0047, respectively, we obtained a good fit of the measured data with the modeled compositions. (b) The measured N_2/He ratios were used to estimate the corresponding CO_2/H_2O ratio (dashed line) returned by the open-system degassing model when the initial magmatic CO_2/H_2O (in equilibrium at 200 MPa) was set at 0.67.

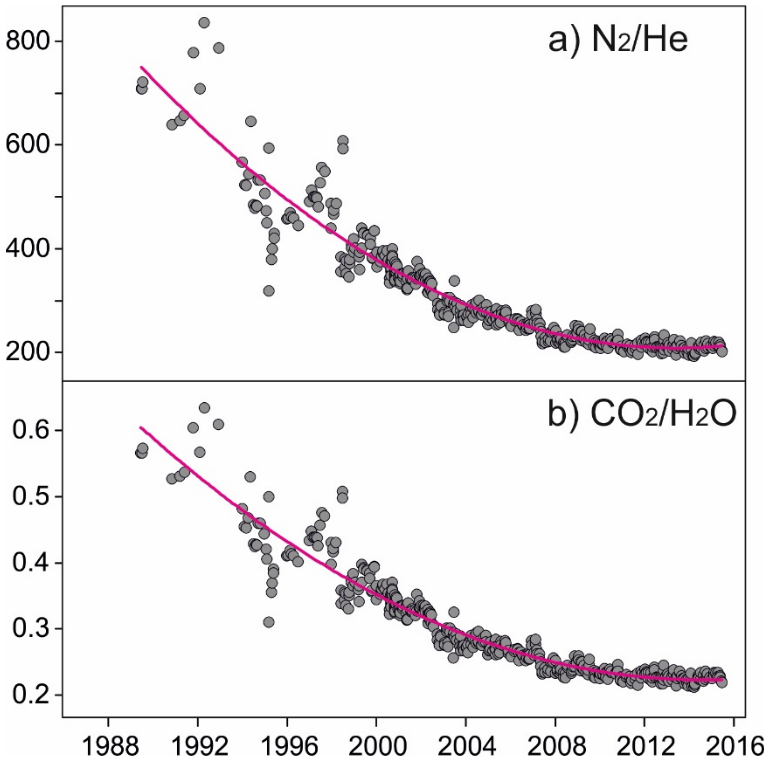

Supplementary Figure 5. CO_2/H_2O ratio of the fluids injected in Simulation 2. In Simulation 2, the measured N_2/He ratios (a) were used to compute the variation of the CO_2/H_2O ratios (b) while considering the relation between the two variables as returned by the theoretical magma-degassing model (Supplementary Fig. 4).

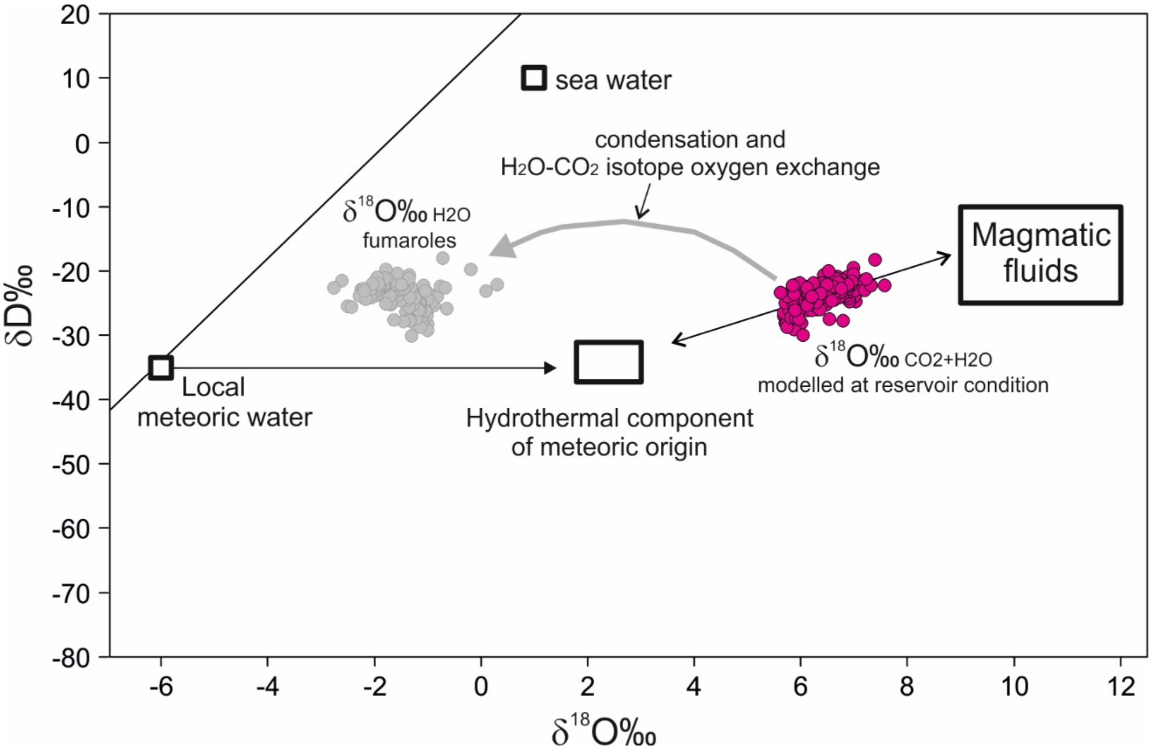

Supplementary Figure 6. δD vs δ^{18}O diagram. Starting from the measured isotopes of fumarolic condensates (post-2000 samples of BG fumaroles, gray circles), the equilibrium δ^{18}O-δD composition of hydrothermal vapor (H_2O+CO_2; red circles) was calculated. Calculations were performed at reservoir temperature (Tc) and CO_2 molar fractions (X_{CO2}), and considering the fractions of condensed steam (*f*) from reservoir to discharge. Tc, X_{CO2} and *f* were estimated applying gas equilibria in the H_2O-H_2-CO_2-CO gas system, following the approach described in ref. 14. Computations involved solving a set of isotope mass balance and fractionation equations. Fractionation during water condensation and H_2O-CO_2 isotope oxygen exchange[51] were taken into account. The re-computed δ^{18}O values refer to the whole CO_2+H_2O system. Based on these model-derived compositions, the isotope signature of CF steam samples is consistent with a mixed meteoric-magmatic origin undergoing condensation.
